# Supplementary material for: Genome-wide identification, characterization and gene expression of BES1 transcription factor family in grapevine (Vitis vinifera L.)
Source: Sci Rep. 2023 Jan 5;13:240. doi: 10.1038/s41598-022-24407-y (PMC9816167; doi:10.1038/s41598-022-24407-y)
Supplement: Supplementary file 3 — Supplementary Information. [file 41598_2022_24407_MOESM3_ESM.zip › plot.pdf]

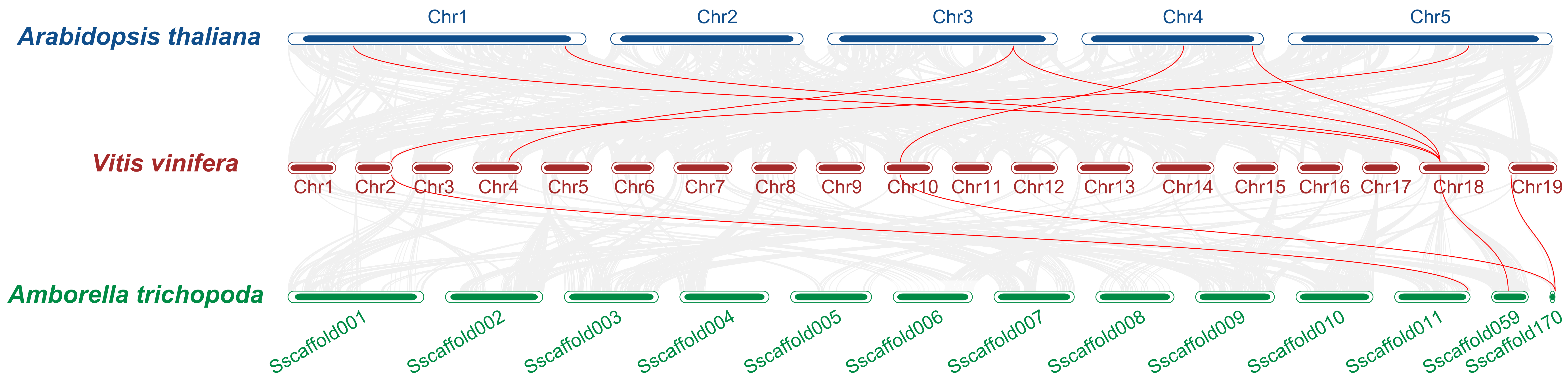
Arabidopsis thaliana
Chr1
Chr2
Chr3
Chr4
Chr5
Vitis vinifera
Chr1
Chr2
Chr3
Chr4
Chr5
Chr6
Chr7
Chr8
Chr9
Chr10
Chr11
Chr12
Chr13
Chr14
Chr15
Chr16
Chr17
Chr18
Chr19
Amborella trichopoda
Sscaffold001
Sscaffold002
Sscaffold003
Sscaffold004
Sscaffold005
Sscaffold006
Sscaffold007
Sscaffold008
Sscaffold009
Sscaffold010
Sscaffold011
Sscaffold059
Sscaffold170
